
JOURNAL INFORMATION
==============================
NLM Title Abbreviation: Wirtschaftsdienst
Journal ID: Wirtschaftsdienst
NLM Journal ID: 101086612

Wirtschaftsdienst (Hamburg, Germany : 1949)

ISSN: 0043-6275

ARTICLE INFORMATION
==============================
PMCID: PMC8684787
PMID: 34955567
DOI: 10.1007/s10273-021-3061-8
Article ID: 3061
Article version: 1
Subjects: Zeitgespräch

Eine höhere Reichweite und heterogene Ausgangslagen erschweren die Vorhersage von Beschäftigungseffekten

Gürtzgen Nicole Nicole.Guertzgen@iab.de

grid.425330.3 0000 0001 1931 2061 Forschungsbereichs A1, Institut für Arbeitsmarkt- und Berufsforschung (IAB) der, Regensburger Straße 104, 90478 Nürnberg, Deutschland
Electronic publication date: 2021 Dec 19
Print publication date: 2021
Volume: 101
Issue: 12
First page: 926
Last page: 929
Copyright: © Der/die Autor:in 2021
License: Open Access: Dieser Artikel wird unter der Creative Commons Namensnennung 4.0 International Lizenz veröffentlicht (https://creativecommons.org/licenses/by/4.0/deed.de).
Open Access wird durch die ZBW — Leibniz-Informationszentrum Wirtschaft gefördert.
License URL: https://creativecommons.org/licenses/by/4.0/

issue-copyright-statement: © The Author(s) 2021

==============================
Prof. Dr. Nicole Gürtzgen ist Leiterin des Forschungsbereichs Arbeitsmarktprozesse und Institutionen am Institut für Arbeitsmarkt- und Berufsforschung in Nürnberg und Professorin für Volkswirtschaftslehre, insbesondere Arbeitsmarktforschung, an der Universität Regensburg.

Seit seiner Einführung im Jahr 2015 wurde der gesetzliche Mindestlohn von anfänglich 8,50 Euro pro Stunde auf inzwischen 9,60 Euro erhöht. Der ursprünglichen Empfehlung der Mindestlohnkommission zufolge sollte der Mindestlohn zum 1. Januar 2022 auf 9,82 Euro und schließlich am 1. Juli 2022 auf 10,45 Euro angehoben werden. In ihrem Koalitionsvertrag haben die Ampelkoalitionär:innen SPD, Grüne und FDP eine Anhebung des Mindestlohns auf 12 Euro in Aussicht gestellt. Offen ist indes noch, wann genau diese Anhebung erfolgen soll. Würden die obigen vorgesehenen Anpassungen noch planmäßig vorgenommen und erfolgte die Erhöhung unmittelbar danach, entspräche die Anhebung gegenüber dem dann zuletzt geltenden Mindestlohn von 10,45 Euro einer Steigerung von ca. 15 %.

## Was wissen wir über die Effekte der Einführung des Mindestlohns?

Um einordnen zu können, welche Folgen eine solch deutliche Anhebung für den Arbeitsmarkt haben kann, bietet sich zunächst ein Blick auf die bisherigen Befunde zur Einführung des gesetzlichen Mindestlohns im Jahr 2015 an. Mit den Auswirkungen der Mindestlohneinführung auf verschiedene Zielgrößen am Arbeitsmarkt hat sich eine Vielzahl von empirischen Studien befasst – darunter auch eine Reihe von Untersuchungen, die die Mindestlohnkommission im Rahmen ihres gesetzlichen Evaluationsauftrags an verschiedene unabhängige Forschungseinrichtungen in Auftrag gegeben hat. Die meisten dieser Studien basieren auf dem Differenz-in-Differenzen-Ansatz (DiD), bei dem die Mindestlohneinführung als natürliches Experiment aufgefasst wird und – vereinfacht gesprochen – die Ergebnisgrößen einer Gruppe von Betroffenen mit denen einer Gruppe von Nichtbetroffenen vor und nach Einführung des Mindestlohns miteinander verglichen werden.[1]

**Prof. Dr. Nicole Gürtzgen** ist Leiterin des Forschungsbereichs Arbeitsmarktprozesse und Institutionen am Institut für Arbeitsmarkt- und Berufsforschung in Nürnberg und Professorin für Volkswirtschaftslehre, insbesondere Arbeitsmarktforschung, an der Universität Regensburg.

## Höhere Entlohnung bei gleichzeitig geringen Beschäftigungseffekten

Da die Einführung des gesetzlichen Mindestlohns nicht zuletzt sozialpolitisch motiviert war, sind seine Wirkungen auf die Entlohnung und Lohnverteilung von besonderem Interesse. Hierfür ist die Eingriffsintensität, also das Ausmaß in dem der Mindestlohn in die bestehende Lohnverteilung eingreift, relevant. Bemisst man die Eingriffsintensität anhand der Zahl der Beschäftigungsverhältnisse, die vor der Mindestlohneinführung unterhalb von 8,50 Euro pro Stunde entlohnt wurden, waren auf Basis der Verdienststrukturerhebung im Jahr 2014 ca. 4 Mio. Beschäftigungsverhältnisse vom Mindestlohn betroffen, was einem Anteil von 11,4 % entspricht (RWI, 2020).

Bezogen auf die Entlohnungseffekte zeichnen die Befunde ein sehr einheitliches Bild: Die meisten Studien belegen, dass der gesetzliche Mindestlohn zu einer Erhöhung der Stundenlöhne am unteren Ende der Lohnverteilung geführt hat und die Lohnungleichheit reduziert hat. In Bezug auf die Monatsverdienste sind die Befunde weniger einheitlich. So gibt es teilweise auch Evidenz dafür, dass höhere Stundenlöhne durch einen Rückgang in den Arbeitsstunden kompensiert wurden.[2]

Da niedrig entlohnte Beschäftigte nur dann vom Mindestlohn profitieren, wenn sie nach Einführung der Lohnuntergrenze weiter beschäftigt bleiben, haben mögliche negative Beschäftigungseffekte des Mindestlohns bereits im Vorfeld seiner Einführung eine breite öffentliche Debat-

1 Einen Überblick über die Literatur liefern etwa Börschlein und Bossler (2019), vom Berge et al. (2020) und Bossler et al. (2020a, b).

2 Siehe hierzu und für die folgende Zusammenfassung der Studienlage den Überblick in Börschlein und Bossler (2019).

te entfacht. Dass diese Debatte sehr kontrovers geführt wurde, hängt vor allem damit zusammen, dass die Richtung der erwarteten Beschäftigungswirkung a priori unklar ist und von der spezifischen Konstellation auf dem Arbeitsmarkt abhängt: Eindeutig negative Beschäftigungseffekte sind dann zu erwarten, wenn der Arbeitsmarkt ein Wettbewerbsmarkt ist und Beschäftigte gemäß ihrer Produktivität entlohnt werden. In diesem Fall senkt ein Mindestlohn die Beschäftigung, sofern er über dem markträumenden Lohn liegt und Arbeitgeber:innen die mindestlohnbedingten Lohnsteigerungen nicht durch Preissteigerungen oder eine höhere Produktivität ausgleichen können. Anders liegt der Fall, wenn der Arbeitsmarkt nicht durch vollkommenen Wettbewerb gekennzeichnet ist. Dies ist etwa in monopsonistischen Arbeitsmärkten relevant, in denen Arbeitgeber:innen Marktmacht ausüben und den Lohn durch den Umfang ihrer Arbeitsnachfrage selbst beeinflussen können. Dieser Lohnsetzungsspielraum kann sich z. B. aus einer mangelnden Mobilität der Beschäftigten ergeben und führt insgesamt dazu, dass Arbeitgeber:innen ihre Beschäftigten unterhalb ihrer Produktivität entlohnen. Auf einem solchen Arbeitsmarkt kann ein Mindestlohn auch zu positiven Beschäftigungseffekten führen, da der so beschriebene Lohnsetzungsspielraum durch die Lohnuntergrenze außer Kraft gesetzt wird.

Insgesamt zeigt sich auf Basis der aktuellen Studienlage, dass die im Vorfeld befürchteten größeren Beschäftigungsverluste nach der Mindestlohneinführung im Aggregat ausgeblieben sind. Um dies zu beurteilen, reicht jedoch nicht ein Blick auf die Entwicklung der sozialversicherungspflichtigen Beschäftigung, die nach der Mindestlohnlohneinführung weiter gestiegen ist. Wie oben angesprochen, erfordert die Quantifizierung der Beschäftigungseffekte vielmehr den Vergleich mit der kontrafaktischen Situation, in welcher der Mindestlohn nicht eingeführt worden wäre. Die vorliegenden Evaluationsstudien, die diesem Umstand Rechnung tragen, weisen mit wenigen Ausnahmen durchgängig sehr geringe negative und teils auch positive Effekte auf die sozialversicherungspflichtige Beschäftigung aus. Negative Effekte konnten jedoch für die Entwicklung der geringfügigen Beschäftigung belegt werden, welche jedoch zu einem großen Anteil in sozialversicherungspflichtige Beschäftigung umgewandelt wurde.

Eine Evaluationsstudie des IAB auf Basis des IAB-Betriebspanels nimmt in diesem Zusammenhang die Anpassungsmechanismen auf der Betriebsebene in den Blick (Bossler et al., 2020a). Den Ergebnissen der Studie lassen sich geringe negative Effekte auf die Beschäftigung nachweisen, die im Wesentlichen auf eine Zurückhaltung bei den Neueinstellungen und weniger auf erhöhte Entlassungen zurückzuführen sind (siehe hierzu auch Bossler und Gerner, 2020). Dies dürfte dem Umstand geschuldet sein, dass Betriebe ihre Beschäftigung aufgrund von Entlassungskosten leichter durch ausbleibende Einstellungen als durch vermehrte Kündigungen anpassen können. Die Studie kommt ebenfalls zu dem Ergebnis, dass die Mindestlohneinführung keine Effekte auf die betriebliche Arbeitsproduktivität entfaltet hat. Bei höheren Lohnkosten und gleichbleibender Arbeitsproduktivität ergeben sich jedoch eindeutig negative Effekte auf die betriebliche Gewinnsituation (Bossler et al., 2020a).

Die Studie belegt zudem, dass sich die Effekte der Mindestlohneinführung in Ost- und Westdeutschland und in Betrieben mit unterschiedlichem Wettbewerbsdruck erheblich unterscheiden. So konzentrieren sich die negativen Beschäftigungseffekte im Wesentlichen auf Betriebe in Ostdeutschland und auf solche, die sich einem hohen Wettbewerbsdruck ausgesetzt fühlen. Ebenso lässt sich zeigen, dass Beschäftigungsverluste in eher monopsonistisch geprägten Branchen, wie dem Hotel- und Gaststättengewerbe und dem Einzelhandel, ungeachtet der hohen Eingriffsintensität des Mindestlohns in diesen Branchen, ausgeblieben sind. Dies stützt die Hypothese, dass ein gesetzlicher Mindestlohn mögliche Lohnsetzungsspielräume von Arbeitgeber:innen, die z. B. aus einer geringen Mobilität der Beschäftigten resultieren, aushebeln kann.

### Implikationen für eine Anhebung des Mindestlohns auf 12 Euro

Welche Schlussfolgerungen lassen sich aus der bisherigen Studienlage für eine Anhebung des Mindestlohns auf 12 Euro pro Stunde ziehen? Zunächst einmal ist ein Blick auf die Reichweite eines gesetzlichen Mindestlohns von 12 Euro hilfreich. Berechnungen auf Basis des SOEP zufolge lässt sich die Eingriffsintensität für alle sozialversicherungspflichtigen oder geringfügigen Hauptbeschäftigungsverhältnisse im Jahr 2019 auf ca. 23 % beziffern (Pusch, 2021). Betrachtet man nur sozialversicherungspflichtige Beschäftigungsverhältnisse, beläuft sich die Eingriffsintensität auf rund 17 %.[3] Diese Berechnungen beziehen sowohl Beschäftigungsverhältnisse, die schon länger andauern, als auch sehr junge Beschäftigungsverhältnisse kurz nach der Neueinstellung mit ein. Da Löhne in der Regel mit zunehmender Betriebszugehörigkeit steigen, ist davon auszugehen, dass der Mindestlohn für Neueinstellungen eine höhere Eingriffsintensität aufweist als für länger bestehende Beschäftigungsverhältnisse. Die Eingriffsintensität bei Neueinstellungen ist von besonderem Interesse, da – wie oben dargelegt – Betriebe ihre Beschäftigung eher

3 Eigene Berechnungen unter Ausschluss der geringfügigen Beschäftigungsverhältnisse auf Basis von Tabelle 1, Pusch (2021).

über eine Zurückhaltung bei den Neueinstellungen als über Kündigungen anpassen. Auf Basis der IAB-Stellenerhebung lässt sich der Anteil der sozialversicherungspflichtigen Neueinstellungen berechnen, welche nach Angaben der befragten Betriebe unterhalb eines Bruttostundenlohns von 12 Euro vorgenommen wurden. Dieser Anteil liegt mit rund 23 % im Jahr 2020 deutlich höher als der Anteil der sozialversicherungspflichtigen Neueinstellungen, der von der Einführung des Mindestlohns im Jahr 2015 betroffen war.[4] Dieser belief sich auf Basis derselben Datenquelle im Jahr 2014 auf knapp 7 % (Gürtzgen et al., 2016).[5] Zu beachten ist, dass die Eingriffsintensität bei Neueinstellungen unter Einbezug geringfügiger Beschäftigungsverhältnisse noch höher ausfallen dürfte – diese Informationen sind jedoch auf Basis der IAB-Stellenerhebung nicht verfügbar. Ebenso werden in der IAB-Stellenerhebung kurzfristige Beschäftigungsverhältnisse tendenziell untererfasst (Gürtzgen und Küfner, 2021). Aus diesem Grunde dürfte die oben ausgewiesene Eingriffsintensität eines Mindestlohns von 12 Euro bei den Neueinstellungen eine Untergrenze darstellen.

## Herausforderungen bei der Vorhersage von Arbeitsmarkteffekten

Dass eine Mindestlohnanhebung auf 12 Euro eine wesentlich größere Reichweite als die anfängliche Mindestlohneinführung in Höhe von 8,50 Euro haben wird, lässt noch keine Aussagen über mögliche Beschäftigungseffekte zu. Auch liefern die oben erwähnten Befunde zu den Effekten der Einführung des Mindestlohns nur eingeschränkt Aufschluss über mögliche Wirkungen auf die zukünftige Beschäftigung. Dies hängt damit zusammen, dass das Gros der empirischen Befunde im Wesentlichen auf sogenannten Ex-Post-Studien basiert. Diese beinhalten eine Evaluation einer bestimmten bereits in Kraft getretenen (daher ex post) Mindestlohneinführung bzw. -erhöhung und ermöglichen somit keine Aussagen über die Effekte zukünftiger hypothetischer Anhebungen. Weiterhin verzichten Ex-Post-Studien auf eine Modellierung der ökonomischen Wirkungszusammenhänge. Deren Berücksichtigung ist jedoch entscheidend, um im Vorhinein – also ex ante – Effekte etwa auf die Beschäftigung abschätzen zu können. Strukturelle Modelle verfolgen demgegenüber das Ziel, zentrale Wirkungskanäle zu modellieren, um auf dieser Basis Ex-ante-Aussagen treffen zu können. Mögliche unterschiedliche Wirkungskanäle lassen sich am Beispiel der Beschäftigungseffekte anschaulich illustrieren: Wie bereits oben dargelegt, kann ein Mindestlohn positive Beschäftigungseffekte entfalten, indem er Friktionen und Marktmacht am Arbeitsmarkt reduziert und dazu führt, dass Arbeitssuchende häufiger für sie akzeptable Jobangebote erhalten als in einer Situation ohne Mindestlohn. Nachteilige Effekte auf die Beschäftigung entstehen dann, wenn Arbeitgeber:innen ihre Arbeitsnachfrage senken oder gar ganz aus dem Markt austreten müssen. Der Nettobeschäftigungseffekt hängt dann davon ab, welcher der beiden Wirkungsmechanismen dominiert. Eine Simulationsstudie auf Basis eines strukturellen Modells, welches diese Transmissionskanäle explizit berücksichtigt, kommt zu dem Ergebnis, dass sich bis zu einem Mindestlohn von 12 Euro beide Effekte in etwa die Waage halten (Blömer et al., 2018). Dieses Ergebnis muss jedoch unter der Einschränkung interpretiert werden, dass als Ausgangslage ein anderer Zeitpunkt, nämlich das Jahr vor der Einführung des Mindestlohns, herangezogen wurde. Zudem wurden in der Simulation zwischenzeitlich aufgetretene makroökonomische Schocks, wie die durch die COVID-19-Pandemie ausgelöste Rezession, sowie Anpassungsreaktionen auf den Mindestlohn etwa in Form von Preiserhöhungen nicht berücksichtigt.

Unter den oben genannten Einschränkungen zeigt die obige Studie ebenfalls, dass die Wirkungen eines einheitlichen Mindestlohns in unterschiedlichen Bereichen des Arbeitsmarkts – differenziert nach Regionen und Berufsgruppen – äußerst heterogen ausfallen werden. So sagen die Simulationen für Ostdeutschland – sowohl für einen Mindestlohn von 8,50 Euro als auch von 12 Euro – anders als für Westdeutschland negative Beschäftigungseffekte voraus. Für die Mindestlohneinführung entspricht dies auch den Ergebnissen der Ex-post-Untersuchungen. Ebenso deuten die Analysen darauf hin, dass ein Mindestlohn von 12 Euro für bestimmte Berufssegmente wie Dienstleistungsberufe eher zu Einbußen bei der Beschäftigung führen wird als für qualifiziertere Berufsgruppen. Dies hängt damit zusammen, dass für Dienstleistungsberufe mögliche positive Beschäftigungseffekte eines Mindestlohns, die durch einen Abbau von Friktionen am Arbeitsmarkt hervorgerufen werden, bereits zu niedrigeren Mindestlöhnen ausgeschöpft sind.

Als Fazit lässt sich festhalten, dass die bislang ausgebliebenen Beschäftigungseinbrüche des im Jahr 2015 eingeführten Mindestlohns wenig Aufschluss über zukünftige Beschäftigungseffekte einer Anhebung des Mindestlohns auf 12 Euro liefern können. Auch die wesentlich höhere Reichweite lässt keine Aussagen darüber zu, wie beschäftigungsschädlich oder -unschädlich die Anhebung ausfallen wird. Zuverlässig vorhersagen lässt sich jedoch,

4 Quelle: IAB-Stellenerhebung, eigene Berechnungen. Die Berechnungen basieren auf den gewichteten Stundenlohn-Informationen zu der letzten sozialversicherungspflichtigen Neueinstellung in der IAB-Stellenerhebung 2014 und 2020 und schließen neu eingestellte Personen unter 18 Jahren aus. Stundenlohnberechnungen auf Basis von Informationen zu monatlichen Gehältern schließen bezahlte Überstunden ein.

5 In dem für das Jahr 2014 berechneten Anteil sind keine Antizipationseffekte bei den Lohnerhöhungen auf das Mindestlohnniveau im Jahr 2014 enthalten, für die die Analyse von Gürtzgen et al. (2016) Hinweise liefert.

dass eine einheitliche Mindestlohnanhebung in unterschiedlichen Segmenten des Arbeitsmarkts differenzierte Wirkungen entfalten wird.

Dass die Ampelkoalitionär:innen das bis dato etablierte Verfahren, nach dem die Mindestlohnkommission auf Basis wissenschaftlicher Beratung eine Empfehlung über die Erhöhung des Mindestlohns abgibt, durch die vorzeitige Anhebung des Mindestlohns auf 12 Euro außer Kraft setzen, ist letztlich eine politische Entscheidung. Für den Arbeitsmarkt stellt die geplante Erhöhung wie bereits schon die Einführung des Mindestlohns ein weitreichendes soziales Experiment dar, dessen Folgen auf Basis angemessener methodischer Instrumente wissenschaftlich zu evaluieren sein werden.



Literatur

Berge, P. vom, J. Beste, M. Bossler, K. Bruckmeier, E.-B. Börschlein und N. Erik-Benjamin (2020), Auswirkungen des gesetzlichen Mindestlohns *Stellungnahme des IAB am 19.3.2020 zur schriftlichen Anhörung der Mindestlohnkommission.
Blömer, M. J., N. Gürtzgen, L. Pohlan, H. Stichnoth und G. J. van den Berg (2018), Unemployment Effects of the German Minimum Wage in an Equilibrium Job Search Model. ZEW Discussion Paper, 2018–032.
Börschlein, E.-B.; Bossler, M. (2019): Eine Bilanz nach fünf Jahren gesetzlicher Mindestlohn: Positive Lohneffekte, kaum Beschäftigungseffekte. IAB-Kurzbericht, 24/2019, Nürnberg.
Bossler M Gerner H-D Employment Effects of the New German Minimum Wage — Evidence from Establishment-level Micro Data Industrial and Labor Relations Review 2020 73 5 1070 1094 10.1177/0019793919889635
Bossler, M., N. Gürtzgen und E.-B. Börschlein (2020a), Endbericht der Studie „Auswirkungen des gesetzlichen Mindestlohns auf Betriebe und Unternehmen“ im Auftrag der Mindestlohnkommission.
Bossler M Gürtzgen N Lochner B Betzl U Feist L The German Minimum Wage: Effects on Productivity, Profitability, and Investments Jahrbücher für Nationalökonomie und Statistik 2020 240 2/3 321 350 10.1515/jbnst-2018-0074
Gürtzgen, N., A. Kubis, M. Rebien und E. Weber (2016), Neueinstellungen auf Mindestlohnniveau: Anforderungen und Besetzungsschwierigkeiten gestiegen, IAB-Kurzbericht, 12/2016.
Gürtzgen, N. und B. Küfner (2021), Hirings in the IAB Job Vacancy Survey and the administrative data — an aggregate comparison, FDZ-Methodenreport, 02/2021.
Pusch, T. (2021), 12 Euro Mindestlohn — Deutliche Lohnsteigerungen vor allem bei nicht-tarifgebundenen Beschäftigten, WSI Policy-Brief, Nr. 62. 10/2021.
RWI (2020), Endbericht der Studie „Auswirkungen des gesetzlichen Mindestlohns auf Löhne und Arbeitszeiten“ im Auftrag der Mindestlohnkommission.
